# Association between serum HE4 and poor periodontal health in adult women

**DOI:** 10.1007/s00784-023-05111-1

**Published:** 2023-06-20

**Authors:** Ruoyan Cao, Shusen Zhang, Jiayu Zhang, Di Miao, Huan Zhou, Yue Chen

**Affiliations:** 1 Key Laboratory of Shaanxi Province for Craniofacial Precision Medicine Research, College of Stomatology, Xi’an Jiaotong University, No. 98, Xiwu Road, Xincheng District, Xi’an, 710004 China; 2 Department of Periodontology, College of Stomatology, Xi’an Jiaotong University, No. 98, Xiwu Road, Xincheng District, Xi’an, 710004 China; 3 Department of Stomatology, Hunan University of Medicine, Hunan, China

**Keywords:** Periodontitis, HE4, NHANES, Cross-sectional study

## Abstract

**Objectives:**

The aim of this study is to explore the association between serum human epididymal protein (HE4) levels and poor periodontal health.

**Materials and methods:**

Data used in our study from the National Health and Nutrition Examination Survey (NHANES) 2001–2002 and Gene Expression Omnibus database (GSE10334 and GSE16134). Periodontitis category was defined by the 2017 classification scheme based on clinical periodontal parameters. Univariate and multivariate logistic regression analyses were used to explore the relationship between serum HE4 levels and the risk of periodontitis. GSEA analysis was performed to investigate the function of HE4.

**Results:**

A total of 1715 adult women over the age of 30 were included in our study. Compared with the lowest tertile, individuals in the highest tertile of HE4 levels were more likely to be Stage III/IV periodontitis (OR_tertile3vs1_ = 2.35, 95% CI: 1.35–4.21). The association was still significant in populations who were less than 60 years old, non-Hispanic white, high school graduate, 1.3 < PI ≤ 3.5, non-smoker, current smoker, non-obese, obese, and who had not diabetes mellitus or had not hypertension. In addition, HE4 expression was upregulated in diseased gingival tissues and involved in cell proliferation and immunity.

**Conclusions:**

Serum HE4 is positively associated with poor periodontal health in adult women.

**Clinical relevance:**

Patients with high serum HE4 levels are more likely to have Stage III/IV periodontitis. HE4 has the potential to be used as a biomarker to predict the severity of periodontitis.

**Supplementary information:**

The online version contains supplementary material available at 10.1007/s00784-023-05111-1.

## Introduction

Periodontitis is a chronic inflammatory disease of the tooth-supporting tissue caused by a variety of pathogenic bacteria, leading to tooth loss [1]. Epidemiological evidence indicates that periodontitis affects approximately 50% of the global population with 10% have serve periodontitis, impairing patient’s quality of life [2, 3]. Thus, it is necessary to explore biomarkers reflecting disease activity to guide clinical decision-making and thereby improve clinical outcomes.

Human epididymal protein 4 (HE4), a secreted glycoprotein encoded by the WFDC2 gene, is widely used as a tumor biomarker for ovarian cancer diagnosis in clinical practice [4]. In addition to being involved in tumor development and progression, HE4 has been implicated in primary Sjögren's syndrome, chronic obstructive pulmonary disease, interstitial lung disease and dilated cardiomyopathy [5–8]. There is evidence that HE4 is involved in extracellular matrix remodeling, and knockdown of HE4 reduces the expression of MMP2 and MMP9 [9, 10]. MMP2 and MMP9 play important roles in the progression of periodontitis. Thus, it is reasonable to speculate that HE4 is associated with periodontal health.

In this study, we found that serum HE4 was associated with poor periodontal health based on the National Health and Nutrition Survey (NHANES; 2001–2002) data. The mRNA expression of HE4 was higher in periodontitis compared with health tissue. We also investigated the function of HE4 based on GSEA analysis in periodontitis. The results of this study highlight the potential application value of HE4 in periodontitis.

## Material and method

### Study design and population

The data used in this cross-sectional study were obtained from the NHANES 2001–2002. The NHANES is a multistage, stratified, clustered probability sampling study focused on the health and nutritional status of civilians in the United States. All participants gave written informed consent and all study protocols were approved by the National Center for Health Statistics (NCHS) Ethics Review Board and was conducted in accordance with the Helsinki Declaration of 1975, as revised in 2013. Participants without a complete full mouth periodontal examination (FMPE), serum HE4 data, and age less than 30 years were excluded. Laboratory data on serum HE4 designed by NHANES 2001–2002 were only available for women, so our study finally included 1715 females.

GSE10334 and GSE16134 were download from Gene Expression Omnibus database (GEO, https://www.ncbi.nlm.nih.gov/geo/). GSE10334 dataset includes 183 periodontitis samples and 64 healthy samples, and GSE16134 dataset includes 241 periodontitis samples and 69 healthy samples. The data of GSE10334 and GSE16134 were based on GPL570 (Affymetrix Human Genome U133 Plus 2.0 Array). The “ComBat” algorithm was applied to correct for batch effects of non-biological technical biases between GSE10334 and GSE16134 dataset.

### Exposure variable

Levels of HE4 were measured based on the Meso Scale Discovery electrochemiluminescence immunoassay platform in the Genital Tract Biology Laboratory (Brigham and Women’s Hospital). The acceptable quality control pool variation was set to 25% and the linearity range of HE4 was 0.59 to 246.34 pmol/l.

### Outcome variable

The outcome of this cross-sectional study was Stage III/IV periodontitis. Periodontal examination included clinical attachment loss (CAL) and probing depth (PD) at six sites per tooth without third molars based on the FMPE protocol. A maximum of 168 sites and 28 teeth per subject could be examined to evaluate periodontal status. The classification system for periodontitis, as specified by the 2017 World Workshop on Periodontal and Peri-Implant Diseases and Conditions, was employed [11]. Periodontitis was diagnosed if the interdental CAL was equal to or greater than 1 mm in at least two non-adjacent teeth, or if the CAL in the buccal/lingual sites was equal to or greater than 3 mm with a PD greater than 3 mm in at least two teeth [12]. Stage I was defined as 1–2 mm CAL, Stage II as 3–4 mm CAL, and Stage III/IV as equal to or greater than 5 mm CAL. Furthermore, to account for management complexity, patients diagnosed with Stage II periodontitis were reclassified as Stage III if their maximum PD measurement was ≥ 6 mm. Additionally, patients diagnosed with Stage III periodontitis were reclassified as Stage IV if they had fewer than 20 teeth (10 pairs) remaining [13].

### Potential confounders

Potential confounders were collected from previous studies, including age, race, marital status, education level, poverty index, smoking status, alcohol consumption, obesity, diabetes and hypertension.

### Gene set enrichment analysis

We assessed the relationship between HE4 and other genes based on spearman correlation test. Then, Gene Set Enrichment Analysis (GSEA) was performed using “clusterProfiler” R packages to explore the function of HE4. The gene sets of “h.all.v7.5.1.entrez” and “c2.cp.kegg.v7.5.1.entrez” were downloaded from the MSigDB database for running GSEA. Adjusted *P* values less than 0.05 were considered statistically significant.

### Statistical analysis

Continuous variables were described as mean ± standard deviation (SD), and categorical variables were presented as absolute frequency and percentage. One-way ANOVA test and Chi-square test were used to assess the differences among the different levels of HE4. Smoothing function was performed to identify any non-linear relationship of serum HE4 levels with Stage III/IV periodontitis. Two multivariable logistic model were applied to explore the association between serum HE levels and poor periodontal health after adjusting for potential confounders. Model I was adjusted for age and race, and model II was additionally adjusted for education level, marital status, PI, obesity, smoking status, alcohol consumption, diabetes and hypertension. In addition, stratified analyses were performed based on all variables in Table 1 to explore the relationship between serum HE4 levels and periodontitis. All the analyses were performed based R software (version 4.1.2). *P* values less than 0.05 were considered significant.

**Table 1:** Characteristics of the participates

Characteristics	Level of HE4			
	Tertile 1 n = 572	Tertile 2 n = 572	Tertile 3 n = 571	*P* value
Age	36.24 ± 12.26	42.58 ± 15.54	53.37 ± 19.40	< 0.001
Age group				< 0.001
≤ 60 years	540 (94.41%)	476 (83.22%)	341 (59.72%)	
> 60 years	32 (5.59%)	96 (16.78%)	230 (40.28%)	
Race				< 0.001
Non-Hispanic White	262 (45.80%)	268 (46.85%)	365 (63.92%)	
Mexican American	143 (25.00%)	154 (26.92%)	95 (16.64%)	
Non-Hispanic Black	114 (19.93%)	95 (16.61%)	80 (14.01%)	
Marital status				< 0.001
Married/living as married	388 (67.83%)	393 (68.71%)	340 (59.54%)	
Never married	109 (19.06%)	79 (13.81%)	67 (11.73%)	
Separated/divorced/widowed	75 (13.11%)	100 (17.48%)	164 (28.72%)	
Education level				0.044
< High school	139 (24.30%)	142 (24.83%)	164 (28.72%)	
High school	131 (22.90%)	126 (22.03%)	150 (26.27%)	
> High school	302 (52.80%)	304 (53.15%)	257 (45.01%)	
PI				0.123
≤ 1.3	137 (23.95%)	140 (24.48%)	137 (23.99%)	
1.3–3.5	218 (38.11%)	199 (34.79%)	227 (39.75%)	
> 3.5	189 (33.04%)	201 (35.14%)	163 (28.55%)	
Obesity				0.018
No	349 (61.01%)	379 (66.26%)	396 (69.35%)	
Yes	215 (37.59%)	182 (31.82%)	162 (28.37%)	
Smoking habit				< 0.001
Non smoker	400 (69.93%)	373 (65.21%)	289 (50.61%)	
Former smoker	96 (16.78%)	124 (21.68%)	117 (20.49%)	
Current smoker	75 (13.11%)	74 (12.94%)	165 (28.90%)	
Alcohol consumption				0.531
Never	113 (19.76%)	122 (21.33%)	116 (20.32%)	
Former	84 (14.69%)	85 (14.86%)	106 (18.56%)	
Mild	147 (25.70%)	161 (28.15%)	154 (26.97%)	
Moderate	104 (18.18%)	94 (16.43%)	81 (14.19%)	
Heavy	86 (15.03%)	75 (13.11%)	84 (14.71%)	
Diabetes mellitus				< 0.001
No	425 (74.30%)	445 (77.80%)	469 (82.14%)	
Yes	37 (6.47%)	40 (6.99%)	68 (11.91%)	
Hypertension				< 0.001
No	450 (78.67%)	418 (73.08%)	291 (50.96%)	
Yes	122 (21.33%)	154 (26.92%)	280 (49.04%)	
Periodontitis				< 0.001
Non-Stage III/IV periodontitis	548 (95.80%)	524 (91.61%)	468 (81.96%)	
Stage III/IV periodontitis	24 (4.20%)	48 (8.39%)	103 (18.04%)	

## Results

A total of 1715 NHANES participants were included in our study, and their baseline characteristics were presented in Table 1. The distributions of education level, PI and alcohol consumption were similar among the three different HE4 levels. Compared with those in the first tertile of HE4 level, individuals in the third tertile tended to be older, non-Hispanic white, separated/divorced/widowed, less educated, non-obesity, current smokers, hypertension and Stage III/IV periodontitis.

**Fig. 1:**
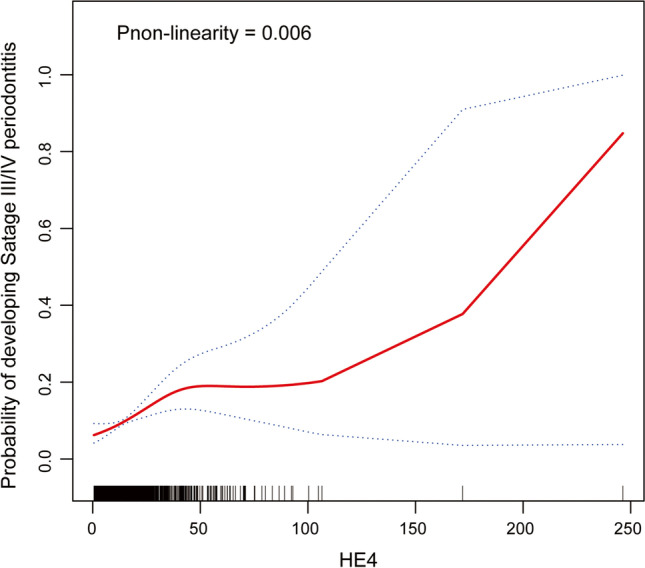
A smooth curve fitting for the association between serum HE4 levels and the risk of Stage III/IV periodontitis

Figure 1 showed a non-linear positive correlation between HE4 and Stage III/IV periodontitis (*P*_non-linearity_ = 0.006). Therefore, we grouped HE4 into tertiles for further analysis. The OR (95% CI) of periodontal disease based on the tertile of HE4 levels were shown in Table 2. High level of HE4 was positively associated with Stage III/IV periodontitis in the different models: Crude model (OR_tertile3vs1_ = 5.03, 95% CI: 3.22–8.14), Model I (OR_tertile3vs1_ = 3.33, 95% CI: 2.05–5.56) and Model II (OR_tertile3vs1_ = 2.35, 95% CI: 1.35–4.21). We also used the CDC/AAP definition [14] to confirm the robustness of the association between HE4 and periodontal status. High level of HE4 increased the risk of moderate/severe periodontitis in the different models: Crude model (OR_tertile3vs1_ = 4.49, 95% CI: 2.82–7.46), Model I (OR_tertile3vs1_ = 3.11, 95% CI: 1.89–5.29) and Model II (OR_tertile3vs1_ = 2.13, 95% CI: 1.21–3.85) (Table S1).

**Table 2:** Association between HE4 and periodontitis

Variable	Crude model		Model I		Model II	
	OR (95%CI)	*P*-value	OR (95%CI)	*P*-value	OR (95%CI)	*P*-value
HE4						
Tertile 1	Ref		Ref		Ref	
Tertile 2	2.09 (1.28, 3.52)	0.004	1.78 (1.07, 3.02)	0.03	1.72 (0.98, 3.10)	0.07
Tertile 3	5.03 (3.22, 8.14)	< 0.0001	3.33 (2.05, 5.56)	< 0.0001	2.35 (1.35, 4.21)	0.003
*P* for trend	< 0.0001		< 0.0001		0.003	

Model I: Adjusted for age and race

Model II: Model I and adjusted for education level, marital status, PI, obesity, smoking status, alcohol consumption, diabetes mellitus and hypertension

The subgroup analysis of the association between HE4 levels and Stage III/IV periodontitis was presented in Table 3. High level of HE4 was positively correlated with Stage III/IV periodontitis in those who were less than 60 years old (OR_tertile3vs1_ = 2.32, 95% CI: 1.25–4.31), high school (OR_tertile3vs1_ = 3.69, 95% CI: 1.30–10.44), 1.3 < PI ≤ 3.5 (OR_tertile3vs1_ = 3.35, 95% CI: 1.36–8.27), separated/divorced/widowed (OR_tertile3vs1_ = 7.82, 95% CI: 1.68–36.45), non-smoker (OR_tertile3vs1_ = 2.42, 95% CI: 1.12–5.25), current smoker (OR_tertile3vs1_ = 3.45, 95% CI: 1.06–11.22), non-obese (OR_tertile3vs1_ = 2.37, 95% CI: 1.13–4.99),obese (OR_tertile3vs1_ = 2.52, 95% CI: 1.10–5.75), who had not diabetes mellitus (OR_tertile3vs1_ = 2.29, 95% CI: 1.27–4.14) or who had not hypertension (OR_tertile3vs1_ = 3.83, 95% CI: 1.74–8.41). In addition, we found that education level, PI, marital status, smoking habit, alcohol consumption and hypertension may impact the relationship between HE4 and periodontitis. However, it is worth noting that the majority of the associations identified in this study share a similar directionality. As such, the clinical implications of these findings may not be significant.

**Table 3:** Effect size of HE4 on periodontitis in each subgroup

Characteristic	HE4			*P* for trend	*P* for interaction
	Tertile 1	Tertile 2	Tertile 3		
Age					0.17
≤ 60	Ref	1.44 (0.78, 2.68)	**2.32 (1.25, 4.31)**	0.12	
> 60	Ref	3.38 (0.81, 14.0)	3.8 (0.96, 15.1)	0.57	
Race					0.09
Non-Hispanic White	Ref	1.57 (0.61, 4.03)	2.38 (0.98, 5.80)	0.04	
Mexican American	Ref	0.89 (0.27, 2.96)	1.74 (0.54, 5.64)	0.24	
Non-Hispanic Black	Ref	2.18 (0.85, 5.61)	2.38 (0.89, 6.39)	0.94	
Education level					0.02
< High school	Ref	1.72 (0.73, 4.08)	2.13 (0.88, 5.11)	0.6	
High school	Ref	1.01 (0.31, 3.29)	**3.69 (1.30, 10.44)**	0.004	
> High school	Ref	2.11 (0.83, 5.37)	1.51 (0.56, 4.04)	0.59	
PI					0.003
≤ 1.3	Ref	1.52 (0.57,4.06)	2.38 (0.87, 6.53)	0.35	
1.3–3.5	Ref	1.43 (0.53, 3.84)	**3.35 (1.36, 8.27)**	0.004	
> 3.5	Ref	2.08 (0.70, 6.20)	1.98 (0.63, 6.21)	0.3	
Marital status					0.01
Married/living as married	Ref	1.22 (0.62, 2.39)	1.91 (0.99, 3.70)	0.05	
Never married	Ref	1.41 (0.24, 8.23)	1.03 (0.13, 8.25)	0.7	
Separated/divorced/widowed	Ref	**6.83 (1.47, 31.78)**	**7.82 (1.68, 36.45)**	0.69	
Smoking habit					0.004
Non smoker	Ref	**2.82 (1.37, 5.82)**	**2.42 (1.12, 5.25)**	0.42	
Former smoker	Ref	0.55 (0.14, 2.10)	1.17 (0.35, 3.88)	0.17	
Current smoker	Ref	1.32 (0.34, 5.11)	**3.45 (1.06, 11.22)**	0.02	
Alcohol consumption					0.03
Never	Ref	**3.68 (1.04, 13.04)**	3.22 (0.85, 12.15)	0.65	
Former	Ref	2.37 (0.62, 9.03)	3.47 (0.96, 12.52)	0.39	
Mild/Moderate/Heavy	Ref	1.09 (0.54, 2.22)	1.72 (0.87, 3.38)	0.12	
Obesity					0.18
No	Ref	2.04 (0.94, 4.40)	**2.37 (1.13, 4.99)**	0.51	
Yes	Ref	1.78 (0.80, 3.99)	**2.52 (1.10, 5.75)**	0.03	
Diabetes mellitus					0.62
No	Ref	1.69 (0.92, 3.08)	**2.29 (1.27, 4.14)**	0.01	
Yes	Ref	2.57 (0.47, 13.96)	5.35 (0.91, 31.60)	0.06	
Hypertension					0.04
No	Ref	1.86 (0.84, 4.09)	**3.83 (1.74, 8.41)**	0.04	
Yes	Ref	1.79 (0.82, 3.90)	1.80 (0.85, 3.83)	0.18	

Adjusted for age, race, education level, marital status, PI, obesity, smoking status, alcohol consumption, diabetes mellitus and hypertension except the subgroup variable

Bold denotes statistical signifcance at *P* < 0.05

**Fig. 2:**
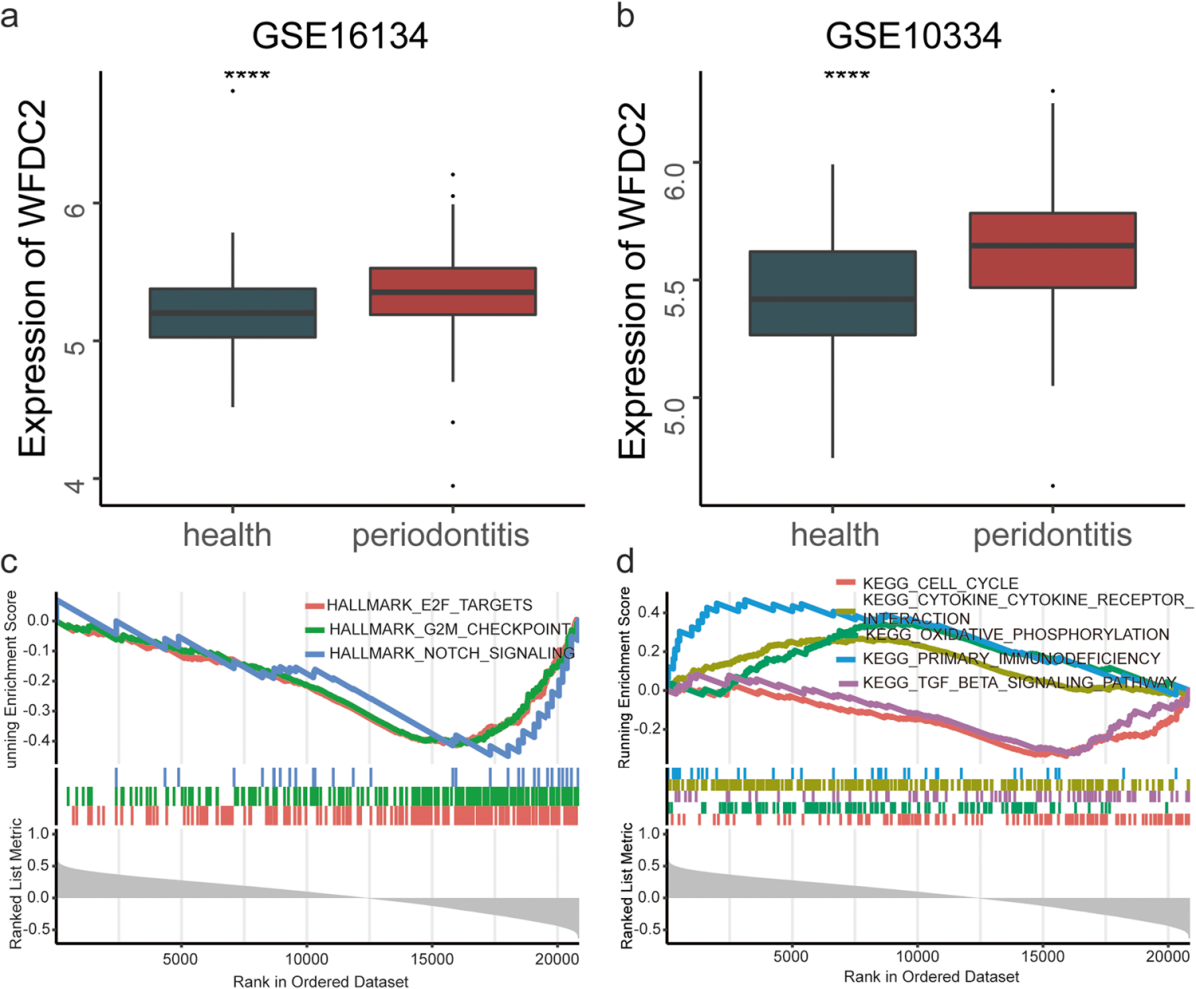
The expression and function of HE4 in periodontitis. (**a**, **b**) Box plot of HE4 expression between periodontitis and health samples in GSE16134 and GSE10334, respectively. (**c**, **d**) GSEA analysis of Hallmarks and KEGG pathway gene sets based on the correlation between HE4 and other genes

We explored the expression of HE4 based on GSE10334 and GSE16134 datasets. The results indicated that HE4 was significantly upregulated in periodontitis samples than periodontally healthy (Fig. 2a, b). Then, we combined GSE10334 and GSE16134 datasets after correcting for batch effects using the “ComBat” algorithm for GSEA analysis. The GSEA analysis indicated that HE4 was negatively associated with E2F targets, G2M checkpoint, cell cycle, notch signaling and TGFβ signaling, while it was positively associated with immune related pathways (primary immunodeficiency, cytokine-cytokine receptor interaction) and oxidative phosphorylation (Fig. 2c, d).

## Discussion

In this cross-sectional study, we found a positive correlation between serum levels of HE4 and Stage III/IV periodontitis in adult women and this relationship remained significant after adjustment for potential confounders. In addition, such association persisted in individuals younger than 60 years old, non-Hispanic white, high school graduate, 1.3 < PI ≤ 3.5, non-smoker, current smoker, non-obese, obese, and who had not diabetes mellitus or had not hypertension. HE4 was upregulated in periodontitis samples and was involved in cell cycle and immune related pathways.

Although HE4 has been found to be associated with multiple diseases, there is limited evidence supporting the association between HE4 and periodontitis. In this study, we have observed that HE4 shows a positive correlation with poor periodontal health, suggesting a dose–response relationship (*P* for trend = 0.003). This could be due to the positive correlation of HE4 with inflammatory mediators such as CRP, IL-6, and IL-8, and the fact that heightened inflammation tends to accelerate the destruction of periodontal tissues. [15]. The role of HE4 in multiple immune-related signaling pathways, including primary immunodeficiency and cytokine-cytokine receptor interaction, has been validated in gingival tissues. Furthermore, HE4 was found to be significantly upregulated in gingival tissues that are affected by periodontitis. In addition to immunity, our findings indicate that HE4 is negatively associated with E2F targets, the G2M checkpoint, and the cell cycle. This suggests that HE4 may inhibit the proliferation of gingival fibroblasts or epithelial cells, leading to an imbalance in periodontal homeostasis. Similarity, HE4 plays an important role in cancer cell proliferation. HE4 inhibits the proliferative capacity of ovarian cancer cells, while enhancing the proliferation of pancreatic and endometrial cancer cells [16, 17].

In our subgroup analysis, we observed that individuals with a high level of HE4 were more prone to developing Stage III/IV periodontitis in all subgroups. Although some associations did not reach statistical significance, this may be owing to reduced statistical power. Among these subgroups, we found a stronger association between HE4 and periodontitis in individuals who do not have hypertension (OR_tertile3vs1_ = 3.83, 95% CI: 1.74–8.41). Systemic inflammation is a link between hypertension and periodontitis, and individuals with hypertension tend to exhibit higher inflammation levels than those without hypertension [18, 19]. Hypertensive patients may therefore have higher tolerance to inflammation due to this increased baseline. Furthermore, HE4 is known to serve a crucial function in inducing inflammatory responses [6, 20]. Therefore, individuals without hypertension with elevated levels of HE4 may experience more significant effects on their periodontal status. However, we have observed that when compared to current smokers in the lowest tertile of HE4, current smokers in the highest tertile of HE4 were 245% more likely to have Stage III/IV periodontitis. Cigarette smoke also increases pro-inflammatory cytokines including IL-1, IL-8, TNF-alpha, IL-6, and GM-CSF, while reducing the levels of anti-inflammatory cytokines like IL-10 [21]. Further studies are needed to explore the mechanisms behind the role of HE4 in individuals who smoke or do not have hypertension.

There are several limitations in our study. First, it is not feasible to identify a causal relationship between HE4 levels and periodontitis based on a cross-sectional design. Second, due to the design of the NHANES 2001–2002 study design on HE4 levels, our study included only female participants. Further research is needed to explore the relationship between HE4 levels and periodontitis in male participants. Finally, we could not rule out all possible residual confounders due to unmeasured confounding factors.

In summary, serum HE4 is positively associated with poor periodontal health in adult women. HE4 is inversely correlated with cell proliferation and positively correlated with immune related signaling pathways.

## Supplementary information

Below is the link to the electronic supplementary material.

Supplementary file1 (DOCX 14 KB)
