# Supplementary material for: Association between serum HE4 and poor periodontal health in adult women
Source: Clin Oral Investig. 2023 Jun 20;27(8):4809–16. doi: 10.1007/s00784-023-05111-1 (PMC10415452; doi:10.1007/s00784-023-05111-1)
Supplement: Supplementary file 1 — Supplementary file1 (DOCX 14 KB) [file 784_2023_5111_MOESM1_ESM.docx]

Table S1 Association between HE4 and periodontitis (CDC/AAP definition)

| Variable | Crude model | |  | Model I | |  | Model II | |
| --- | --- | --- | --- | --- | --- | --- | --- | --- |
|  | OR (95%CI) | *P*-value |  | OR (95%CI) | *P*-value |  | OR (95%CI) | *P*-value |
| HE4 |  |  |  |  |  |  |  |  |
| Tertile 1 | Ref |  |  | Ref |  |  | Ref |  |
| Tertile 2 | 1.68 (0.98, 2.93) | 0.06 |  | 1.40 (0.82, 2.44) | 0.23 |  | 1.33 (0.74, 2.45) | 0.35 |
| Tertile 3 | 4.49(2.82, 7.46) | < 0.0001 |  | 3.11 (1.89, 5.29) | < 0.0001 |  | 2.13 (1.21, 3.85) | 0.01 |
| *P* for trend | < 0.0001 | |  | < 0.0001 | |  | 0.01 | |
